# Tapeworm Enigma

**DOI:** 10.3201/eid2806.AC2806

**Published:** 2022-06

**Authors:** Byron Breedlove, Richard Bradbury

**Affiliations:** Centers for Disease Control and Prevention, Atlanta, Georgia, USA (B. Breedlove);; Federation University Australia, Berwick, Victoria, Australia (R. Bradbury)

**Keywords:** art science connection, emerging infectious diseases, art and medicine, about the cover, tapeworm enigma, William C. Campbell, parasitology, helminths, neglected tropical diseases, lymphatic filariasis, onchocerciasis, dracunculiasis, Nobel Prize, ivermectin, tapeworms

**Figure:**
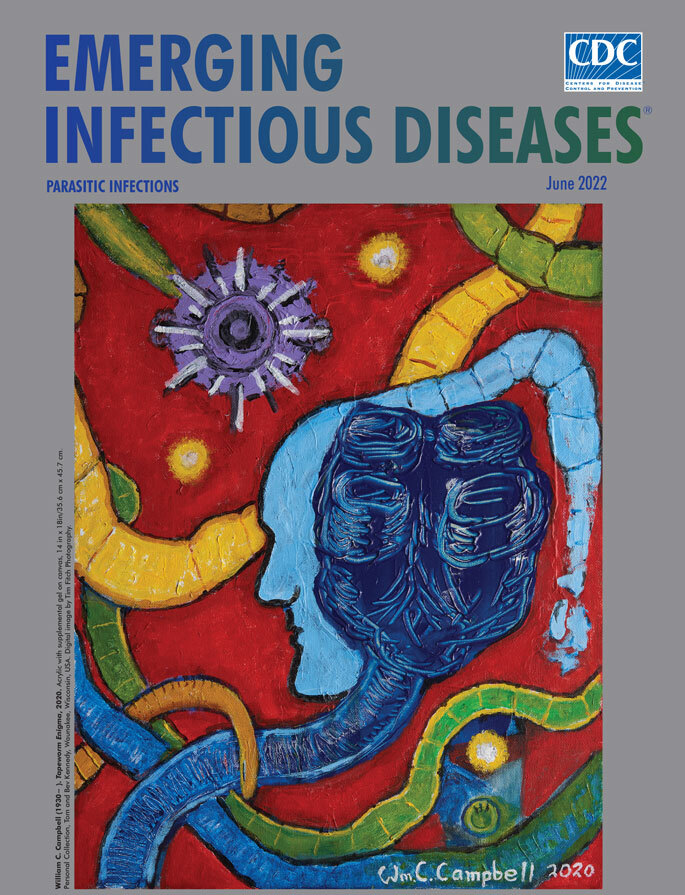
**William C. Campbell (1930−), *Tapeworm Enigma*, 2020**. Acrylic with supplemental gel on canvas, 14 in x 18 in/35.6 cm x 45.7 cm. Personal Collection, Tom and Bev Kennedy, Waunakee, Wisconsin, USA. Digital image by Tim Fitch Photography.

Among the parasitic organisms that can transmit infections to humans are about 300 species of helminths (parasitic worms). In his book *People, Parasites, and Plowshares,* Columbia University parasitologist Dickson Despommier notes, “These are the most underappreciated of our parasites that have for centuries kept a low profile, cruising just under the radar screen of the world’s health agencies.”

Helminths of three main groups—cestodes (tapeworms), trematodes (flatworms, or flukes), and roundworms (nematodes)—are human parasites responsible for an enormous burden of disease. A number of helminthic infections are designated as neglected tropical diseases. Among those diseases are lymphatic filariasis, caused by threadlike worms spread by mosquitoes; onchocerciasis or river blindness, caused by the parasitic worm *Onchocerca volvulus* and transmitted from bites by black flies; and dracunculiasis, commonly called Guinea worm disease, transmitted by ingestion of water fleas*.*

A trio of soil-transmitted helminths—intestinal roundworms (*Ascaris lumbricoides*), whipworms (*Trichuris trichiura*), and hookworms (*Necator americanus*, *Ancylostoma duodenale*, and *Ancylostoma ceylanicum*)―are dubbed the “unholy trinity.” Those helminths infect humans via ingestion of food or water contaminated with soil containing their eggs or larvae or, in the case of hookworms, may infect by direct passage of their larvae through the skin. According to the Centers for Disease Control and Prevention, a large portion of the world’s population is infected with one or more of those parasitic worms: 807−1,121 million people with roundworms, 604−795 million with whipworms, and 576−740 million with hookworms.

In his book, Despommier explains that tapeworms, among the most well-known parasitic helminths, “get their common name from their off-putting resemblance to a white cloth tape measure.” He adds that “judging by the global distribution of tapeworm infections, and the number of different host species that harbor them, parasitologists now consider the cestodes to be among the most successful of the worm infections.” 

The most common way that humans acquire taeniasis—intestinal infection with tapeworms of the genus *Taenia* and caused by worms of three species, *Taenia solium* (pork tapeworm), *Taenia saginata* (beef tapeworm), and *Taenia asiatica* (Asian tapeworm)—is by eating raw or undercooked pork or beef containing tapeworm cysts. According to the World Health Organization, among those tapeworms, the most debilitating health problems are caused by *T. solium*.

This month’s cover art, *Tapeworm Enigma*, is one of a number of paintings created by Irish-American biologist and parasitologist William C. Campbell. Campbell studied at Trinity College at the University of Dublin and the University of Wisconsin, Madison, Wisconsin, USA. After graduating, he joined the Merck Institute for Therapeutic Research in New Jersey, where he worked for the next 33 years, also holding adjunct professorships at the University of Pennsylvania, New York Medical College, and Drew University. 

Campbell’s research on ivermectin as an antihelmintic treatment for animals led to the discovery that this agent would also be effective in treating onchocerciasis. As a result, in 2015 half of the Nobel Prize in Physiology or Medicine was awarded jointly to Campbell and Satoshi Ōmura for their co-discovery of ivermectin, currently widely used against nematode parasites, including the etiologic agents of river blindness, elephantiasis, and strongyloidiasis. (Although other applications for ivermectin are being applied in many parts of the world, the focus of this essay is parasitic diseases and the use of ivermectin as an antihelmintic drug.) 

Campbell is also a poet and a painter. His persistent curiosity and extensive knowledge about parasitic worms are apparent in his artistic endeavors. In a 2019 newspaper interview, Campbell explained, “Some people focus on one subject and then have a hobby to sort of escape from their main subject. I don't look at it that way at all. I'm not painting pictures to escape from the science that I do. I want to bring the science with me into the painting and bring the two together.”

*Tapeworm Enigma* exemplifies his surrealistic approach to painting. A moment is needed for the viewer to realize that the segmented strands of green, blue, yellow, and orange that cover the vibrant red background indeed represent tapeworms. A purple rostellum, streaked with white and black, seems disturbingly agile, all too eye-catching. At the bottom left of the painting is the orange segmented body of a *Dibothriocephalus latus* worm tapering into the unsegmented neck and terminating in its distinctive scolex, with a slit-like groove for attachment to the intestine. The light blue profile of a human face juxtaposed with the dark blue scolex of a *T. saginata* tapeworm in the center of the painting invariably forces the viewer to pause and contemplate, possibly while also squirming a bit, the parasite–host relationship. 

Campbell (pers. comm, email, 2022 April 7) explained that “The painting features the attachment-organ of the beef tapeworm of humans (dark blue and highly textured). Adjacent to it is a human profile that gives way to a tapeworm (light blue) that disintegrates, as tapeworms do, as it gets beyond maturity. In upper left is a stylized ‘*en face*’ view of the pork tapeworm. Elsewhere are stretches of nondescript tapeworms. Less easily found is a larval stage (hexacanth embryo) of a tapeworm, as well as suggestions of the fish tapeworm of humans.” 

Exercising his prerogative as an artist, Campbell concludes, “The ‘meaning’ of the picture must remain cryptic!” Perhaps, though, he offered a clue in a 2017 interview in the *Irish America Magazine*: “I consider them [parasitic worms] beautiful,” he said. “They are just doing their own thing and not meaning to be destructive. And I have said in some recent papers that the objective is not to get rid of parasitic worms, the objective is to get rid of parasitic diseases.”
